# Utilization of Erector Spinae Plane Block in the Chronic Pain Clinic for Two Patients With Post-Thoracotomy Pain

**DOI:** 10.7759/cureus.8988

**Published:** 2020-07-03

**Authors:** Jamal Hasoon, Ivan Urits, Omar Viswanath, Musa Aner

**Affiliations:** 1 Department of Anesthesia, Critical Care and Pain Medicine, Beth Israel Deaconess Medical Center and Harvard Medical School, Boston, USA; 2 Department of Anesthesia, Critical Care and Pain Medicine, Beth Israel Deaconess Medical Center, Harvard Medical School, Boston, USA; 3 Pain Management, Valley Pain Consultants - Envision Physician Services, Phoenix, USA

**Keywords:** erector spinae plane block, neuropathic pain, chronic pain, regional anesthesia, post-thoracotomy pain

## Abstract

The erector spinae plane (ESP) block is a regional block that has become more commonly utilized in the setting of acute pain and post-operative analgesia. This block has been successfully utilized for pain management after a variety of surgical procedures for immediate post-operative pain management. This block is now gaining more utilization in the chronic pain setting for neuropathic pain conditions. We describe the use of this block at our pain clinic for the treatment of two patients with refractory neuropathic pain after thoracotomy as well as video-assisted thoracic surgery (VATS). Our cases further demonstrate the utility of this block for long-term pain control of neuropathic pain conditions, especially post-thoracotomy pain.

## Introduction

The erector spinae plane (ESP) block is a regional block that can be utilized to provide analgesia for a variety of pain conditions. The block spreads medication along the fascial plane and has been primarily utilized for acute post-operative pain control [1-3]. The use of this technique for pain management has recently started to expand for a variety of neuropathic pain conditions [4-8]. Patients who undergo thoracotomy or video-assisted thoracic surgery (VATS) are at risk for developing chronic neuropathic pain after their surgeries, and this pain is commonly undertreated [9]. We describe our experience utilizing this block in two patients with severe neuropathic pain after surgery refractory to medication management.

## Case presentation

The first patient was an 83-year-old male who underwent placement of endovascular grafts for a descending thoracic aortic aneurysm. The patient had two grafts placed at the time of initial repair. This was complicated by a type Ib endoleak one week later requiring repair and placement of an additional graft. He also required a VATS washout of a hemothorax at the time of the replacement. After recovery and discharge from the hospital, the patient was referred to the chronic pain clinic for severe burning left-sided chest wall pain along the VATS incision sites. The patient reported the pain was a constant burning pain that occasionally felt stabbing in nature for short periods of time during stretching and quick movements. The pain was rated as 10/10 intensity on a numerical rating scale (NRS). The pain interfered with his ability to do any meaningful activities and also disrupted his sleep. He had trialed acetaminophen, lidocaine patches, and neuropathic medications with no relief. The patient was counseled regarding the risks and benefits of the ESP block and consented to proceed with the procedure.

The second patient was a 48-year-old male who underwent prior thoracotomy for a previous thoracic trauma. The patient presented to the pain clinic for assistance with his pain management after several failed treatment attempts by medication management. The patient endorsed severe burning and stabbing pain along the incision site that was constant and unrelenting in nature. He reported 10/10 pain intensity on NRS. The patient had trialed acetaminophen, nonsteroidal anti-inflammatory drugs (NSAIDs), lidocaine patches, neuropathic agents, and antidepressants without relief. He reported that the only thing that provided him with moderate relief was the continued use of oxycodone. This patient was also counseled regarding the risks and benefits of the ESP block and consented to proceed with the procedure.

**Figure 1:**
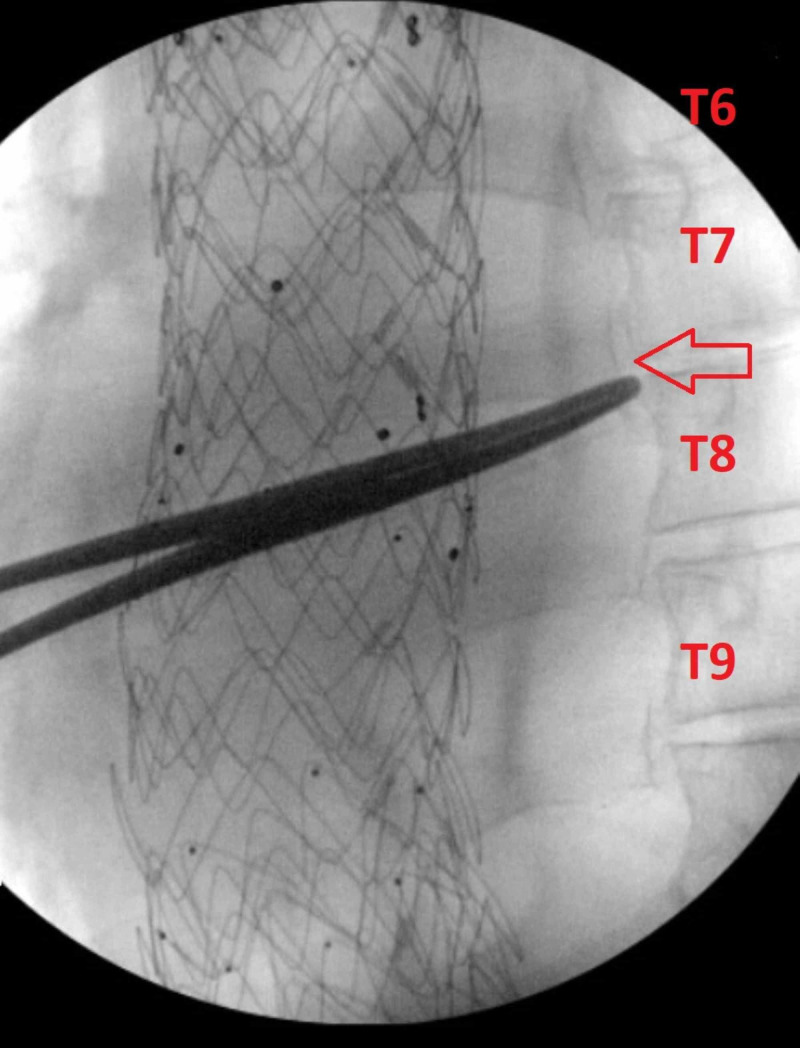
Fluoroscopic confirmation Fluoroscopy was first used to confirm the correct levels for the block. The red arrow is pointing towards the transverse process, which is the target for this block.

Both patients underwent the procedure with fluoroscopic and ultrasound guidance at the T7-T8 levels using an 18 gauge, sonovisible needle. The patients first underwent fluoroscopy to confirm the appropriate level that would be targeted (Figure 1).

**Figure 2:**
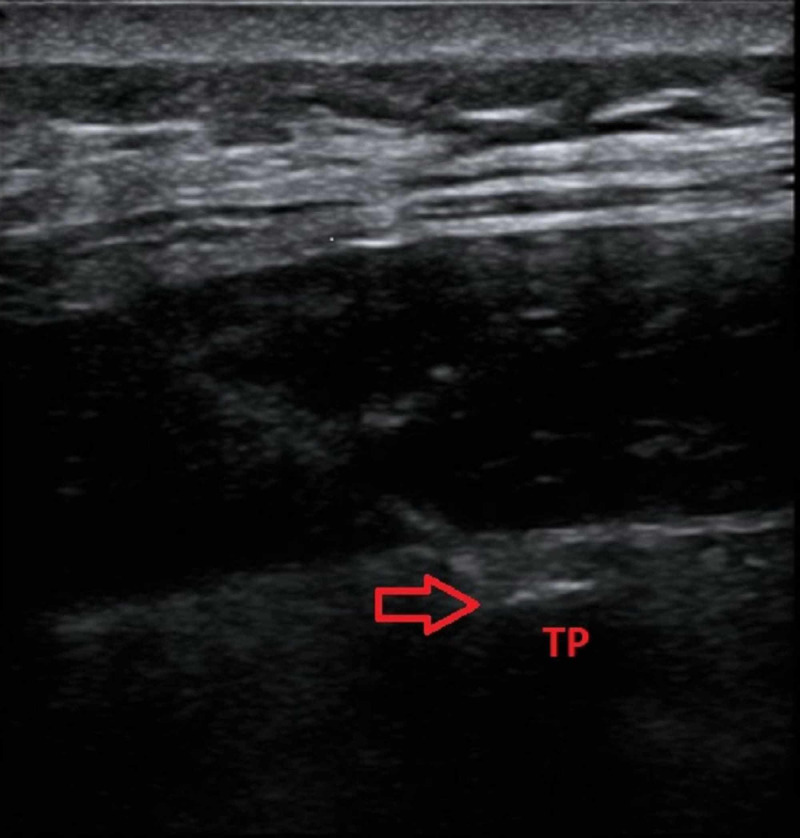
Erector spinae plane block with ultrasound The block was performed under ultrasound guidance. The red arrow highlights needle placement on the transverse process (TP).

The solution used for both patients included 1 mL of methylprednisolone 40 mg/mL, with 9 mL of 0.25% bupivacaine for a total of 10 mL volume for the blocks. The blocks were performed under ultrasound guidance using an in-plane technique that allowed for good visualization of local anesthetic lifting the erector spinae muscle off of the tip of the transverse process (Figure 2). Both procedures were performed without complications.

## Discussion

The first patient reported almost immediate resolution of his pain symptoms in the recovery area. He reported he had absolutely no pain at the VATS incision sites and this pain relief lasted through his one-month follow-up appointment. At his follow-up appointment, he continued to endorse complete pain relief and was not using any medications at that time. The second patient reported 90% relief shortly after the injection. At his one-month follow-up appointment, he continued to endorse 50% pain relief at that time and had weaned his opioid use by 50% as well. Both patients reported satisfaction with the results of the procedure. 

Patients who undergo thoracotomy or VATS are at risk for developing chronic debilitating neuropathic pain after their surgeries, and these patients are often undertreated [9]. The ESP block has been successfully utilized for acute post-operative pain management after a variety of surgical procedures and can be used for chronic neuropathic pain conditions. We have demonstrated the successful use of this block at our pain clinic for the treatment of two patients with refractory neuropathic pain. This regional technique is safe, easy to perform, and well tolerated by patients. We believe physicians with an interest in chronic pain conditions should consider this technique in their treatment algorithms for patients suffering from chronic neuropathic chest wall pain conditions

## Conclusions

The ESP block can be performed under ultrasound guidance in the clinic setting and can be utilized for both acute and chronic pain conditions. Physicians should consider this technique for patients with chronic chest wall pain, post-thoracotomy pain, and intercostal neuralgia unresponsive to previous injections. Our case further demonstrates the usefulness of this block for pain control in patients with chronic neuropathic conditions related to thoracotomy pain.
